# Molecular Determinants of *in vitro* Plant Regeneration: Prospects for Enhanced Manipulation of Lettuce (*Lactuca sativa* L.)

**DOI:** 10.3389/fpls.2022.888425

**Published:** 2022-05-09

**Authors:** Tawni Bull, Richard Michelmore

**Affiliations:** ^1^The Genome Center, University of California, Davis, Davis, CA, United States; ^2^Graduate Group in Horticulture and Agronomy, University of California, Davis, Davis, CA, United States; ^3^Department of Plant Sciences, University of California, Davis, Davis, CA, United States

**Keywords:** regeneration, organogenesis, lettuce, somatic embryogenesis, WUSCHEL, *Lactuca sativa* (L.)

## Abstract

*In vitro* plant regeneration involves dedifferentiation and molecular reprogramming of cells in order to regenerate whole organs. Plant regeneration can occur via two pathways, *de novo* organogenesis and somatic embryogenesis. Both pathways involve intricate molecular mechanisms and crosstalk between auxin and cytokinin signaling. Molecular determinants of both pathways have been studied in detail in model species, but little is known about the molecular mechanisms controlling *de novo* shoot organogenesis in lettuce. This review provides a synopsis of our current knowledge on molecular determinants of *de novo* organogenesis and somatic embryogenesis with an emphasis on the former as well as provides insights into applying this information for enhanced *in vitro* regeneration in non-model species such as lettuce (*Lactuca sativa* L.).

## Introduction

**FIGURE 1:**
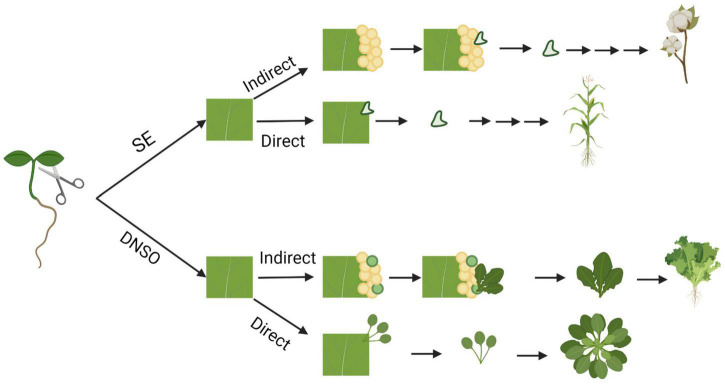
Pathways of *in vitro* regeneration of vascular plants. Somatic embryogenesis (SE) and *de novo* shoot organogenesis (DNSO) can occur directly on the explant or indirectly with the formation of pluripotent callus as an intermediate step. Species that are capable of regeneration for each pathway are represented from top to bottom: cotton, maize, *Arabidopsis*, and lettuce. Figure created using BioRender (https://biorender.com/).

Plants have evolved a remarkable ability to regenerate tissues from differentiated organs, which involves the conversion of one cell type to others. Such plasticity provides the ability to regenerate whole organs and plants via dedifferentiation of cells and reprogramming of cell fates. There are three main types of regeneration: (1) Tissue regeneration, (2) *de novo* organogenesis, and (3) somatic embryogenesis (Xu and Huang, 2014; Sugimoto et al., 2019). Bryophytes have high capacity for tissue regeneration; for example, *Marchantia* spp. are capable of regenerating new meristems within their thallus (Yasui et al., 2019). However, vascular plants follow different regeneration pathways, which include *de novo* organogenesis or somatic embryogenesis (Figure 1). *De novo* organogenesis involves the regeneration of whole organs that did not previously exist. There are two types of *de novo* organogenesis: direct and indirect regeneration. Direct regeneration involves the development of organs directly from explants, whereas indirect regeneration involves an intermediate undifferentiated callus phase. For example, some plants, such as *Jatropha curcas* and succulents of the Cactaceae and Crassulaceae families (Preece, 2003; Severino et al., 2011), are capable of direct regeneration of new roots and shoots from stem cuttings. In contrast, many plants, such as lettuce, exhibit indirect organogenesis and regenerate shoots from calli (Michelmore and Eash, 1985). Somatic embryogenesis involves the regeneration of embryo or embryo-like structures from somatic cells, which can develop into a whole plant. In all forms of regeneration, cells must undergo dedifferentiation or transdifferentiation (reprogramming) into a more totipotent cell, ultimately changing the fate of the progenitor cell.

Plant tissue culture and totipotency were first proposed by Haberlandt in 1902 (Krikorian and Berquam, 1969; Thorpe, 2007), who attempted to culture isolated photosynthetic leaf cells. Although this proved unsuccessful, it was the start of many decades of work on developing and improving plant tissue culture methods for multiple plant species. These failed experiments led to the development of root cultures using root tip cells in tomato and bud cultures. In 1904, embryo culture was first successful when embryos of crucifers (Brassicaceae) were isolated aseptically and grown in culture (Norstog, 1979). The first “true” plant tissue cultures were obtained on Knop’s medium from cambial tissues of sycamore maple (*Acer psuedoplatanus*) by Gautheret in 1934. This approach was optimized by additions of auxin, indole acetic acid (IAA), and B vitamins. This resulted in tissues that could be grown indefinitely in culture and the regeneration of both roots and shoots (Gautheret, 1934, 1935, 1939). The previous studies, however, used explant tissues that already contained meristematic cells. It was not until 1948 that methods were developed to induce roots and shoots from non-meristematic explants (Skoog and Tsui, 1948). This drastically increased the number of species that could be studied using *in vitro* culture systems (Miller et al., 1955; Skoog and Miller, 1957), and led to the recognition of the importance of exogenous ratios of cytokinin and auxin in culture medium. The differing ratios were shown to affect cell fate transition to either rooting or shooting from callus cells (Skoog and Miller, 1957), where high ratios of auxin to cytokinin promoted root regeneration, low ratios of auxin to cytokinin promoted shoot regeneration, and intermediate levels promoted proliferation of callus tissues. From the early to mid-1900s, research helped develop common plant tissue culture methods and media still used today (van Overbeek et al., 1941; Gautheret, 1942, 1955; Nobe’court, 1955). The earliest plant tissue culture media were based on nutrient necessities of whole plants, with the most common being Knop’s solution (Loomis and Schull, 1937). Numerous studies were conducted to optimize culture medium and in 1962, Murashige and Skoog reported a medium (MS salts) containing salt concentrations 25 times higher than those in Knop’s solution; in particular this resulted in much higher concentrations of NO3− and NH4+. The development of MS salts is still considered to be a major breakthrough in tissue culture because MS salts are still commonly used in plant tissue culture. The combination of exogenous plant hormones and appropriate salts allowed the study of basic plant biology questions about cell behavior, genetic improvement, disease biology, germplasm conservation, and clonal propagation.

Plant tissue culture to achieve *in vitro* regeneration was originally used to answer fundamental questions in plant biology but has since evolved to be foundational for genetic improvement, micropropagation, genetic engineering, and biotechnology (Michelmore et al., 1987; Zhang et al., 2006; Loberant and Altman, 2010; Xu and Huang, 2014; Chokheli et al., 2020). However, *in vitro* regeneration is not possible for all plant species and regeneration is very genotype dependent. Therefore, studying the molecular determinants of plant regeneration and exploiting these signaling pathways for improved *in vitro* regeneration of those recalcitrant genotypes and species is important. This review provides a synopsis of our current understanding of the pathways involved in *de novo* organogenesis and somatic embryogenesis. We focus on what is known of the molecular determinants of indirect *de novo* shoot organogenesis, which is the mode of regeneration in lettuce (*Lactuca sativa* L.). Finally, we describe future directions for improvement of *in vitro* regeneration of lettuce and other Compositae species.

## Molecular Determinants of Regeneration

Recently, many advances have been made toward understanding the cellular and molecular mechanisms that underlie plant regeneration (Xu and Huang, 2014; Ikeuchi et al., 2016; Sugimoto et al., 2019). Each of the regeneration processes described above have been studied in detail in model plants such as *Arabidopsis thaliana*. Each process entails a complex of molecular players involved in signaling and developmental pathways that regulate the dedifferentiation (somatic embryogenesis) or reprogramming (*de novo* organogenesis) of cells.

### Organogenic Callus Formation

Callus formation is the first step in indirect organogenesis. Based on morphology, calli are thought to be the result of the dedifferentiation of cells to form totipotent cells. Callus can originate from the initiation of multiple pathways that contain some overlap in gene expression (Fehér, 2019) and can be auxin or wound induced (Fehér, 2019). In *Arabidopsis*, auxin induced calli resemble pluripotent cells similar to root tip cells at the molecular level and originate from pluripotent pericycle cells located adjacent to xylem poles (Atta et al., 2009; Sugimoto et al., 2010; Fehér, 2019). Root cell-like, auxin-induced callus follows a similar pathway as lateral root formation. In contrast, wound-induced callus does not involve players of lateral root formation, but rather occurs via upregulation of cytokinin signaling (Iwase et al., 2011a,b; Ikeuchi et al., 2017). Due to the similarity of gene expression patterns during callus formation with other developmental pathways some consider it a form of transdifferentiation rather than dedifferentiation (Fehér, 2019).

**FIGURE 2:**
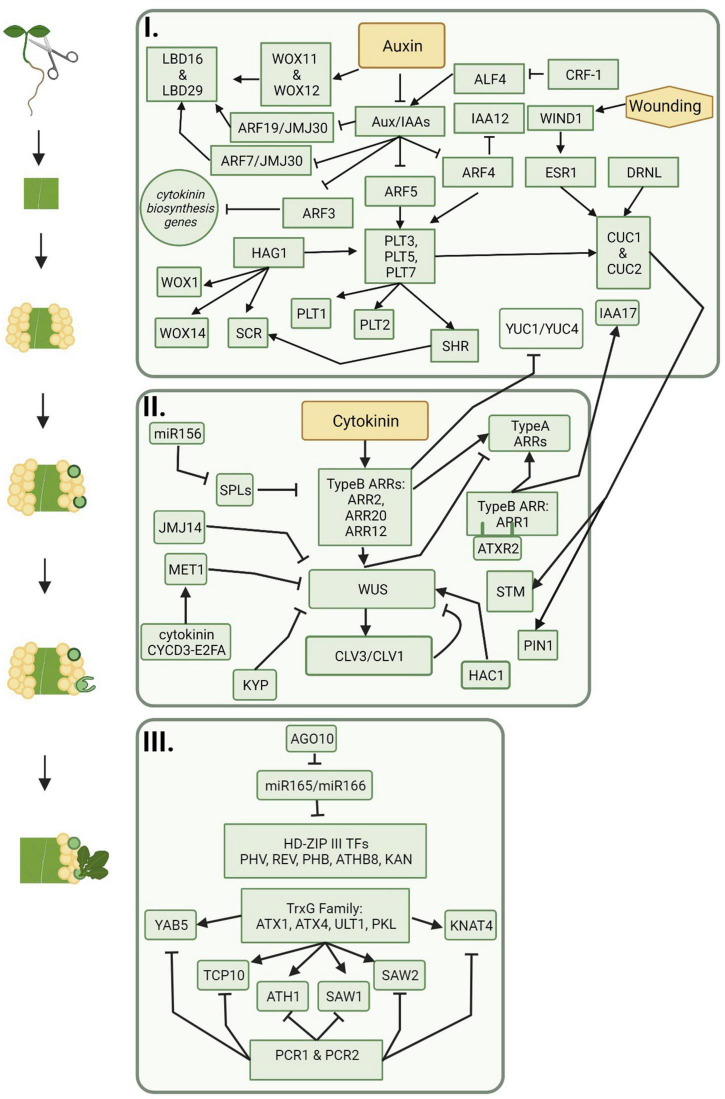
The progression of molecular players during indirect *de novo* shoot organogenesis. Callus is formed on auxin rich medium and includes signaling pathways represented in box one. Shoot promersitems and meristematic centers are formed on cytokinin rich medium and include signaling pathways represented in box two. Shoot regeneration follows meristem formation and is represented by the signaling pathways included in box three. Figure created using BioRender (https://biorender.com/).

Many genes and transcription factors that are involved in lateral root development are also critical players in auxin-induced callus formation (Figure 2). For example, the *LATERAL ORGAN BOUNDARIES* (*LBD*) family of genes, such as *LBD16*, *17*, *18*, and *29*, are critical to both lateral root formation and callus production (Fan et al., 2012; Feng et al., 2012; Xu et al., 2012; Lee H.W. et al., 2019). Ectopic expression of *LBD* genes led to the spontaneous formation of callus without exogenous applications of auxin and cytokinin, and repression of *LBD16* showed inhibited callus formation (Fan et al., 2012). In lateral root formation, *LBD16* and *LBD29* are positively regulated by AUXIN RESPONSE FACTOR7 (ARF7) and ARF19, which provides evidence that *ARFs* are also involved in callus formation (Okushima et al., 2007). Furthermore, JUMONJI C DOMAIN CONTAINING PROTEIN 30 (JMJ30) interacts with ARF7 and ARF19 and directly binds to cis elements of *LBD16* and *LBD29*, promoting their expression (Lee et al., 2018). Other key players in both lateral root and callus formation are *ABERRANT LATERAL ROOT FORMATION 4* (*ALF4*) and *SOLITARY ROOT/IAA14* (*SLR/IAA14*). *ALF4* is involved in the earliest divisions of pericycle cells during lateral root formation. In *alf4* mutants, callus-forming capability was lost in multiple tissues (DiDonato et al., 2004; Sugimoto et al., 2010). It was later shown that *ALF4* is targeted for downregulation by CALLUS FORMATION RELATED*-*1 (CRF-1), which encodes an enzyme involved in very long chain fatty acid (VLCFA) biosynthesis (Shang et al., 2016). Another molecule involved in VLCFA biosynthesis is the AP2 transcription factor, PUCHI, which is also a key regulator controlling cell proliferation in lateral root primordia; *puchi-1* mutants resulted in both defective and disorganized lateral root and callus formation further indicating a link between these pathways (Trinh et al., 2019). SLR is a member of the auxin signaling protein family Aux/IAA, and *slr-1* mutants in *A. thaliana* were defective in both lateral root and callus formation (Fukaki et al., 2002; Shang et al., 2016). The functions of these genes and transcription factors provides evidence that callus formation and lateral root development have similar underlying mechanisms. In addition, callus formation can be initiated via a wound-induced signaling pathway and activation of a cytokinin response. Transcription factors involved during wound-induced callus formation include APETALA2/Ethylene Responsive Factor (AP2/ERF)-type transcription factors, WOUND-INDUCED DEDIFFERENTIATION1 (WIND1), and homologs (Iwase et al., 2011a,b, 2013). In Arabidopsis, expression of *WIND1* and homologs are upregulated upon wounding and promote pluripotent callus formation at cut sites (Iwase et al., 2011a,b). Expression of *Arabidopsis WIND1* was also shown to induce callus formation in other species such as rapeseed, tomato, and tobacco (Iwase et al., 2013). A transcriptome analysis showed WIND1 activates over 2,000 genes involved in multiple pathways including wound-induced cellular reprogramming and vascular formation (Iwase et al., 2021).

Among the genes upregulated by WIND1 are those encoding for other AP2/ERF-type transcription factors including *PLETHORA* (PLT) genes (Kareem et al., 2015; Iwase et al., 2021). *PLT* genes work through the auxin signaling pathway, are often transcribed in response to auxin accumulation, and are activated downstream of *ARF7* and *ARF19* (Aida et al., 2004; Hofhuis et al., 2013). *PLT3*, PLT5, and PLT7 upregulate *PLT1* and *PLT2*, which are important players in the regulation of lateral root formation, root apical meristem maintenance (RAM), and callus pluripotency (Xu et al., 2006; Durgaprasad et al., 2019). In *Arabidopsis, PLT1* is also upregulated by JANUS through the recruitment of RNA Polymerase II to the root meristem (Xiong et al., 2020). In addition to root meristem maintenance, PLT proteins play important roles in conjunction with *BABYBOOM/PLT4* (*BBM/PLT4*) in early embryogenesis (described further in section “Somatic Embryogenesis”), and activate regeneration responses in shoot organs by regulating the shoot promoting factors *CUPPED-SHAPED COTYLEDON1 (CUC1)* and *CUC2* (Radhakrishnan et al., 2020). PLT-CUC2 together work through the auxin biosynthesis pathway and are essential for proper distribution and repolarization of auxin through PIN-FORMED (PIN) proteins (described further in section “*De novo* Root Organogenesis”) (Kareem et al., 2015; Shimotohno et al., 2018; Radhakrishnan et al., 2020).

Callus formation also involves epigenetic regulators. One regulator, HISTONE ACETYLTRANSFERASE OF THE GNAT/MYST SUPERFAMILY 1 (HAG1), also known as A. thaliana GENERAL CONTROL NON-REPRESSED 5 (AtGCN5), acts upstream of *PLT1* and *PLT2* (Kornet and Scheres, 2009; Kim et al., 2018). HAG1 also epigenetically upregulates root meristem genes *WUSHCEL RELATED HOMEOBOX 5* (*WOX5*), *WOX14*, and *SCARECROW* (*SCR)* by acetylating the N terminus of histone 3. HAG1 is further involved in determining the root–shoot axis in embryo development and is a regulator of floral meristem activity (Kim et al., 2018). The RAM gene, *ROOT CLAVATA-HOMOLOG 1* (*RCH1*), is also highly expressed in callus (Sugimoto et al., 2010), providing further evidence of homologies between lateral root development and callus formation. Although initiation of callus can follow multiple pathways, this provides further evidence that each pathway contains overlapping players.

### *De novo* Root Organogenesis

*De novo* root organogenesis is the process by which adventitious roots are formed from detached plant tissues such as leaves and stems. Multiple studies have investigated the regeneration of the RAM in *A. thaliana* (Tian et al., 2002; Casamitjana-Martínez et al., 2003; Galinha et al., 2007; de Smet et al., 2008; Müller and Sheen, 2008; Perilli et al., 2012). The quiescent center (QC) is the site of stem cell maintenance of the RAM that is regenerated after QC ablation or entire removal of the root tip; polar transportation of auxin driven by PIN-FORMED (PIN) proteins results in auxin accumulation in cells adjacent to the damaged QC cells, which drives the reprogramming to new QC cells (van den Berg et al., 1997; Wildwater et al., 2005).

One of the key molecular players in root organogenesis is auxin. In *Arabidopsis*, auxin accumulates at cut sites, which induces expression of the homeobox transcription factors WOX11 and WOX12 (Liu et al., 2014). WOX11 and WOX12 directly upregulate *WOX5*, *LBD16*, and *LBD29*, marking the first step in cell differentiation and the formation of root meristems (Goh et al., 2012; Liu et al., 2014; Hu and Xu, 2016). Auxin accumulation at wound sites in *Arabidopsis* drives the expression of *PLT* genes (as seen in callus formation), which will in turn upregulate *SHORT ROOT* (*SHR*) (Kareem et al., 2015). The SHR proteins will localize to the nucleus, inducing the expression of *SCR*; SHR and SCR are both involved in QC identity and radial patterning (van den Berg et al., 1997; Wildwater et al., 2005). SCR and PLT work together with plant-specific teosinte-branched cycloidea PNCP (TCP) in PLT-TCP-SCR complexes to promote the organization of PIN proteins and expression of *WOX5* in new meristem QC cells (Xu et al., 2006; Shimotohno et al., 2018). Root primordia formation is inhibited in *shr, plt1*, and *plt2* mutants, indicating that these genes play an important role during root formation from root founder cells (Bustillo-Avendaño et al., 2018).

### *De novo* Shoot Organogenesis

Shoot organogenesis may occur with direct regeneration of a shoot from an explant or indirect regeneration from a callus (Figure 1). Because a callus seems to resemble root tip cells rather than shoot cells at the molecular level, callus cells must undergo changes in gene expression that push the cells toward shoot development rather than root development. Shoot regeneration has been studied extensively in plants; however, while many genes and hormones have been identified as important players in the process (Figure 2), the detailed molecular interactions and pathways are unclear (reviewed in Xu et al., 2006; Su and Zhang, 2014; Xu and Huang, 2014; Ikeuchi et al., 2016; Lardon and Geelen, 2020).

**FIGURE 3:**
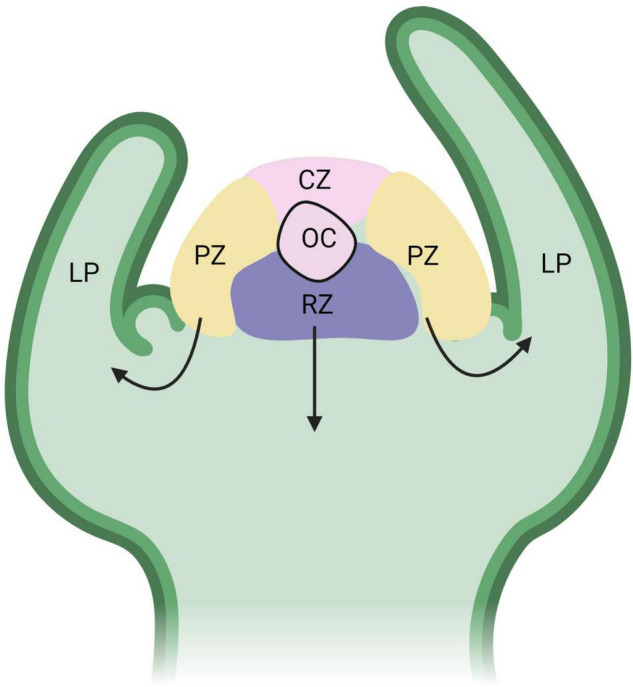
Functional domains of the shoot apical meristem (SAM). The organizing center (OC) is part of the central zone (CZ), which consists of a stem cell pool that replenishes cells to the peripheral zone (PZ) and rib zone (RB). The black arrows represent the direction of differentiating cells from the PZ to form leaf primordia (LP) and the RZ to form the stem. WUS expression is high in the OC and is regulated by CLV3/CLV1 from the CZ in a negative feedback loop. Figure created using BioRender (https://biorender.com/).

Regeneration of shoots from callus requires the formation of a primary meristem or a shoot apical meristem (SAM) (Figure 3). Similar to the RAM, the SAM contains a population of pluripotent stem cells that give rise to all aboveground organs of a plant. The undifferentiated state of the organizing center (OC), which is similar to the RAM QC, and surrounding stem cells is maintained by a feedback mechanism between WUSHEL (WUS) and the signaling peptide CLAVATA3 (CLV3) (Sarkar et al., 2007). Leaves and other lateral organs arise from the peripheral regions of the SAM and the stem arises from the basal cells, called the rib zone. The SAM also contains the central zone, which consists of a stem cell pool that will replenish cells in the peripheral and rib zones that have further differentiated (Bowman and Eshed, 2000; Kwiatkowska, 2004). Unlike auxin accumulation in the RAM, the SAM contains high levels of cytokinins. Organization of auxin and cytokinin in cells help promote differentiation of pluripotent cells to either shoot or root cells.

Shoot regeneration from callus occurs in four stages: (1) Formation of a pluripotent callus, (2) shoot promeristem formation, (3) shoot progenitor development, and (4) shoot regeneration (Shin et al., 2020). The development of a pluripotent callus cell mass (section “Synopsis of Studies on the Regeneration of Lettuce”) that highly expresses the No Apical Meristem/*A. thaliana* activating factor (NAC) transcription factor genes, *CUC1* and *CUC2* (Gordon et al., 2007), transitions into promeristems composed of a primary meristem of actively dividing cells. Within the callus *CUC2* expression marks pre-meristematic regions by promoting cell proliferation and leading to the localized upregulation of a key shoot meristem regulator, *SHOOT MERSITEMLESS* (STM), and *PIN1*. As seen in *de novo* root organogenesis, PIN1 proteins polarly localize, denoting areas of cellular reprogramming toward promeristematic cells (Gordon et al., 2007). Both STM and PIN1 aid in the development of radial patterning as STM marks the promeristem and PIN1 marks primordia (Gordon et al., 2007). Because PIN1 proteins are important players in both promeristem formation and root *de novo* organogenesis, this suggests that auxin transport is important for both shoot and root meristem patterning.

Proper regulation and distribution of *CUC1, CUC2*, and *WUS* are critical for shoot progenitor cells. These NAC transcription factors are subject to upstream regulation during shoot promeristem formation. AP2/ERF-type transcription factors, ENHANCER OF SHOOT REGENERATION 1 (ESR1)/DORNROSCHEN (DRN) and ESR2/DRN-LIKE (DRNL) participate in upstream regulation of *CUC* genes by actively binding to the promoter and inducing expression (Banno et al., 2001; Kirch et al., 2003; Ikeda et al., 2006). Mutants of *esr1, esr2*, and *esr1 esr2* show a reduction in shoot regeneration. This is likely due to improper regulation of *CUC1* and *CUC2* (Matsuo et al., 2011). WIND1 also upregulates *ESR1* by directly binding to the vascular-responsive motifs in the *ESR1* promoter (Iwase et al., 2017), suggesting that WIND1 is important in multiple plant regeneration processes. PLT5 and PLT7, which are induced during callus production, also influence the expression of *CUC1* and *CUC2* (Kareem et al., 2015). This further suggests that the molecular players and pathways involved in shoot regeneration are intertwined.

WUSCHEL (WUS) is a key regulator of the SAM and is upregulated during shoot regeneration. Expression of *WUS* is an important part of the conversion of a promeristem to a shoot progenitor as it represses cell division, cell elongation, and auxin-induced expression. This directs cell fate toward shoot development rather than root development. Ectopic expression of *AtWUS* results in *de novo* meristem formation and organogenesis in multiple plant species including *Arabidopsis* (Gallois et al., 2002; Negin et al., 2017), rice (Victorathisayam and Sridevi, 2020), and cotton (Bouchabké-Coussa et al., 2013). *WUS* expression is restricted to high cytokinin domains, while *CUC2* expression tends to be restricted to low cytokinin and high auxin domains. This is consistent with high expression of *CUC2* during induction of callus on media using higher concentrations of auxin (Daimon et al., 2003; Kareem et al., 2015). Regulation of *WUS* is subject to epigenetic regulation. METHYLTRANSFERASE1 (MET1), KRYPTONITE (KYP), histone acetyl transferase1 (HAC1), and JMJ14 are all required for proper expression of *WUS*, SAM organization, and shoot development (Li et al., 2011; Ishihara et al., 2019). *MET1* is induced by the cytokinin-CYCD3-E2FA module, which represses *WUS* expression, allowing cells to retain callus identity rather than transitioning to shoot cells. However, in later stages of *de novo* shoot organogenesis, *MET1* is spatially regulated, allowing for an increase in *WUS* expression in the inner cell layers of the callus (Liu et al., 2018). Previously, it was thought that *WUS* expression in the inner callus cell layers is directly activated by the cytokinin-responsive Type B ARABIDOPSIS RESPONSE REGULATORS (ARRS), ARR1, ARR2, ARR10, and ARR12 (Dai et al., 2017). However, a recent study showed that ARR1 is a strong inhibitor of callus formation and shoot regeneration. This occurs through indirect repression of *CLV3* by competitive binding with ARR12 (Liu et al., 2020). ARR1 also indirectly represses *WUS* by inducing expression of the auxin response repressor gene *INDOLE-3-ACETIC ACID INDUCIBLE17* (*IAA17*) (Liu et al., 2020). In addition, Type-B ARRs negatively regulate the expression of the auxin biosynthetic genes *YUCCA1* (*YUC1*) and *YUC4* (Meng et al., 2017). This results in indirect upregulation of *WUS* expression. Although it has been known for decades that auxin and cytokinin signaling is important for plant regeneration, these findings further untangle the underlying mechanisms of the signaling pathways.

Eukaryotic stem cells tend to have open chromatin states, while differentiated cells tend to have closed chromatin states (Shchuka et al., 2015). Epigenetic controls include Trithorax group (trxG) and Polycomb Group (PcG) proteins. The *A. thaliana* trxG, ATXR2, interacts with ARR1 and methylates the Type A ARRs, *ARR5* and *ARR7*, marking them for increased transcription. This leads to a repression of cytokinin signaling and a reduction in *de novo* shoot organogenesis (Lee et al., 2021). PcG protein complexes, specifically POLYCOMB REPRESSIVE COMPLEX1 (PRC1) and PRC2, are chromatin modifiers and bind to Polycomb Response Elements (PRE) to keep genes transcriptionally repressed in order to fine-tune the balance between cell proliferation and cell differentiation (Köhler and Hennig, 2010). PRC2 suppresses leaf identity via H3K27me3 of leaf identity genes. PRC2 is also involved in callus formation as PRC2 mutants *curly leaf swinger (clf swn)* and *embryonic flower2 (emf2)* are incapable of developing callus from leaf and cotyledon explants but retained the ability to form callus in root explants (He et al., 2012). This suggests PCR2 represses leaf identify genes, allowing for the transition to root-like callus cells. Other instances of epigenetic regulation during the early stages of regeneration include gene priming by LYSINE-SPECIFIC DEMETHYLASE 1-LIKE 3 (LDL3), which involves the elimination of methylation of lysine 4 on histone 3 (H3K4me2) during callus formation. This indirectly promotes the expression of genes that are involved in shoot progenitor development (Ishihara et al., 2019).

Regulatory microRNA, miR156, plays a role in activating cytokinin signaling by targeting *SQUAMOSA PROMOTER BINDING PROTEIN-LIKE* (*SPL*). *SPL* genes control transitions in shoot development—juvenile-to-adult and vegetative-to-reproductive—by binding to and regulating Type-B ARRs, decreasing shoot regenerative capacity with age (Xu et al., 2015, 2016). miRNA156 expression is higher in younger tissues, which partially explains why younger explant tissue (i.e., cotyledons) is more amenable to *in vitro* regeneration, when compared to more mature tissue types. Type B ARRs and WUS also regulate the Type A ARRs, *ARR7* and *ARR15*, which negatively regulate cytokinin signaling (Buechel et al., 2010).

After proper development of shoot progenitor cells, activation of leaf identity genes will lead to the development of leaf tissues and leaf emergence. Two important players involved in shoot regeneration are miR165 and miR166, both of which target HD-ZIP III transcription factor genes *PHABULOSA* (*PHB*), *PHAVOLUTA* (*PHV*), *REVOLUTA* (*REV*), *KANADI* (*KAN*), and *ARABIDOPSIS THALIANA HOMEOBOX GENE 8* (*ATHB8*) (Shin et al., 2020). *PHB, PHV, REV*, and *KAN* function in radial leaf patterning (abaxial vs. adaxial), and *phb*, *phv*, *rev*, and *kan* mutants show a transition of abaxial leaf fates into adaxial leaf fates as well as altered auxin gradients (McConnell et al., 2001; Emery et al., 2003; Zhou et al., 2019). *ATHB8* and *SHR* expression activate simultaneously and lead to leaf vein precursor cells (Gardiner et al., 2010). An RNA-induced silencing complex, ARGONAUTE10 (AGO10), helps sequester and repress the activity of miR165 and miR166. This indirectly promotes the activity of these leaf identity genes. Interestingly, accumulation of miR165/166 in overexpressing *Arabidopsis* mutants resulting in less HD-ZIP III transcription factor activity in shoot progenitor cells, increased the overall shoot regeneration (Xue et al., 2017). This suggests that leaf identity genes work to suppress *in vitro* transition from meristematic cells into shoot cells. In addition, *AGO10* is repressed by LBD12, resulting in reduced apical meristem size (Ma et al., 2017). Leaf identity genes are also subject to epigenetic regulation. TrxG proteins, ATX1, ATX4, ULTRAPETALA1 (ULT1), and PICKLE (PKL), act as antagonists of PCR1 and PCR2 to activate transcription of leaf identity genes, which will aid in the development of leaves from shoot progenitor cells (Köhler and Hennig, 2010). In *A. thaliana*, ATX4 protein tri-methylates histone 3 (H3K4me3) to increase the expression of the shoot identity genes *ARABIDOPSIS THALIANA HOMEOBOX GENE 1* (*ATH1*), *KNOTTED1-LIKE HOMEOBOX (KNOX) GENE 4* (*KNAT4*), *SAWTOOTH 1* (*SAW1*), *SAW2*, *TCP FAMILY TRANSCRIPTION FACTOR 10* (*TCP10*), and *YABBY 5* (*YAB5*) (Lee K. et al., 2019).

As elaborated above, *de novo* shoot regeneration is controlled by a complex network of genetic and epigenetic factors. Although we are gaining a more detailed understanding of the molecular players involved in this network via forward and reverse genetic approaches, there is clearly more information to discover involving interactions between these genetic, epigenetic, and hormone signaling pathways.

### Embryogenic Callus Formation

Formation of embryogenic callus results from acquisition of a new cell fate through expression of embryonic regulators. Similar to organogenic calli, embryogenic calli have been observed to originate from cells surrounding vascular tissue (pre-procambial cells) (de Almeida et al., 2012). Endogenous application of plant growth regulators such as auxin and cytokinin have been shown to induce proliferation of embryonic tissues in some species, such as soybean and cotton (Raza et al., 2020; Elhiti and Stasolla, 2022). This is similar to auxin-induced callus formation suggesting upregulation of *ARFs* such as *ARF7* and *ARF19* are also requirements for the formation of embryonic callus. Furthermore, *LEAFY COTYLEDON1* (*LEC1*) and *LEC2* genes are major embryonic regulators that control embryo maturation and development (Gaj et al., 2005). *LEC1* overexpression induced embryogenic callus formation in *Arabidopsis*; however, *lec1* and *lec2* mutants resulted in the development of fewer somatic embryos via only indirect somatic embryogenesis (Gaj et al., 2005). This suggests that *LEC1* is sufficient, but not necessary to the formation of embryogenic callus. Overexpression of the MADS-box transcription factor, AGAMOUS-LIKE 15 (AGL15), induced embryogenic callus-like structures on SAMs and extended embryonic development from callus in *Arabidopsis* (Harding et al., 2003). Expression of specific genes and presence of proteins have been observed in embryogenic callus, but not observed in non-embryogenic callus. The MADS-box gene, *CUS1*, whose amino acid sequence is highly similar to Arabidopsis AGL1 and AGL5 amino acid sequences, was detected in embryogenic callus during somatic embryogenesis in cucumber (Filipecki et al., 1997). Additionally, in sugar cane, unique proteins were identified during embryonic callus formation including proteins related to metabolic activity and stress (Schuabb Heringer et al., 2015). Induction of somatic embryogenesis and formation of proembyrogenic masses on calli (section “Somatic Embryogenesis”) involves different molecular players than formation of promeristems during organogenesis, but differences between embryogenic calli and organogenic calli formation, if any, are still not well characterized.

### Somatic Embryogenesis

A second type of *in vitro* regeneration is somatic embryogenesis. Somatic embryogenesis results when a differentiated somatic cell undergoes molecular changes and genetic/epigenetic reprogramming resulting in the formation of a bipolar somatic embryo. In tissue culture, somatic embryogenesis can be induced in response to the addition of exogenous plant growth regulators or the introduction of stressful conditions. Similar to *de novo* organogenesis, somatic embryogenesis may originate directly at wound sites of explants or indirectly from embryogenic callus (Quiroz-Figueroa et al., 2006). Species tend to regenerate either through *de novo* organogenesis (e.g., tomato, lettuce, pepper) or somatic embryogenesis (e.g., cotton, wheat, rice) but rarely both (e.g., chickpea, purple coneflower) (Ozias-akins and Vasil, 1982; Michelmore et al., 1987; Rueb et al., 1994; Murthy et al., 1996; Choffe et al., 2000; Leelavathi et al., 2004; Heidmann et al., 2011; Sun et al., 2015).

Regulators and genetic determinants of embryo initiation are not well understood, although auxin signaling and accumulation are thought to play a major role. In tissue culture, addition of auxin is used to induce somatic embryogenesis by exposure of explants to high levels of auxin immediately followed by a transfer to auxin-free medium (Méndez-Hernández et al., 2019). This allows for the formation of auxin gradients within the developing embryos—areas of high auxin promote *WUS* expression, which denote areas of future SAM development as mentioned previously (Ikeuchi et al., 2016). In *Arabidopsis*, several *ARFs* are both up and downregulated during the first steps of somatic embryogenesis, and multiple *arf* mutants showed inhibited somatic embryogenesis (Wójcikowska and Gaj, 2017). *SOMATIC EMBRYOGENESIS RECEPTOR-LIKE KINASES* (SERKs), specifically *SERK1* in *Arabidopsis*, are upregulated in embryonic callus and are continually expressed from megasporogenesis until the heart stage of the embryonic development (Hecht et al., 2001). Other genes, such as auxin-responsive gene *EgIAA9* from *Elaeis guineensis*, have been shown to be upregulated during somatic embryogenesis initiation (Ooi et al., 2012).

The transcription factor BABY BOOM (BBM) and the LEC1-AB13-FUS3-LEC2 (LAFL) complex are master regulators of somatic embryogenesis (Horstman et al., 2017; Jones et al., 2019). *BBM* encodes an AINTEGUMENTA-LIKE (AIL) AP2/ERF and directly regulates all LAFL genes. *LAFL* genes are also regulated by a BBM-like protein, PLT2 (Horstman et al., 2017). The *LAFL* gene group consists of the *LEC* transcription factor genes, including *LEC1*, *LEC2*, and *FUSCA3* (*FUS3*), and the ABA signaling transcription factor, ABSCISIC ACID INSENSITIVE 3 (ABI3). Somatic embryogenesis events are shown to significantly decrease in *lec* mutants (Gaj et al., 2005), and the overexpression of *LEC2* led to an increase in the expression of auxin biosynthesis genes *IAA30*, *YUC2*, *YUC4*, and *YUC10* (Stone et al., 2008; Junker et al., 2012), suggesting that *LEC* genes and the LAFL complex help promote auxin activity. LEC2 also induces the expression of *LEC1*, *LEAFY COTYLEDON 1-LIKE (L1L)*, *ABI3*, and *FUS3.* Another transcription factor, AGL15, has been shown to directly regulate *LAFL* (Zheng et al., 2009) and promote the expression of the AP2/ERF gene *At5g61590* (Zheng et al., 2013). *At5g61590* is a relative of the *Medicago truncatula SOMATIC EMBRYO-RELATED FACTOR 1* (*MtSERF1*), which is essential for somatic embryogenesis (Mantiri et al., 2008). Recently, another MADS-box transcription factor, AGL18, was identified as an active regulator in somatic embryogenesis in *Arabidopsis* (Paul et al., 2022). Overexpression of *AGL18* resulted in an increase in somatic embryogenesis, while a decrease was observed in *agl18* mutants; *agl15 agl18* double mutants resulted in even less frequent development of somatic embryos. While the functions of AGL15 and AGL18 transcription factors were relatively redundant, different gene targets for each transcription factor were present and an AGL15/AGL18 regulatory loop was identified. This provides evidence that AGL18 may act in conjunction with AGL15 during somatic embryogenesis. Along with *BBM*, *LAFL*, and *AGL15*, the ectopic expression of *WUS*, *PLT4*/*BBM*, *PLT5*/*EMBRYMAKER*, *MYB118*, and *RWP*-*RK DOMAIN*-*CONTAINING4* (*RKD4*)/*GROUNDED* (*GRD*) leads to the induction of somatic embryogenesis in *Arabidopsis* (Lotan et al., 1998; Boutilier et al., 2002; Harding et al., 2003; Gallois et al., 2004; Waki et al., 2011).

The master regulators work with other transcription factors to balance auxin, gibberellin (GA), and abscisic acid (ABA) signaling. In particular, the balance of GA and ABA has a major role in controlling cell identity in the developing embryo. Embryonic cells have been shown to have a higher ratio of GA to ABA than somatic cells (Yamaguchi et al., 2001; Mitchum et al., 2006; Hu et al., 2008). The LAFL transcription factors, LEC1, LEC2, FUS3, and AGL15, downregulate GA biosynthesis genes (Curaba et al., 2004; Zheng et al., 2009), while FUS3 positively regulates the ABA pathway (Gazzarrini et al., 2004). LEC1 and LEC2 promote the expression of auxin biosynthesis genes (Braybrook et al., 2006; Junker et al., 2012), and AGL15 negatively regulates the auxin response genes, *ARF6*, *ARF8*, and *TRANSPORT INHIBITOR RESPONSE1* (*TIR1*) (Zheng et al., 2016). LEC1 and AGL15 positively regulate *ABI3.* Generally, these transcription factors work to negatively regulate GA biosynthesis and positively regulate ABA and auxin biosynthesis, transitioning cells from embryonic cells (high GA/ABA ratios) into differentiated somatic cells (low GA/ABA ratios). MYB-family transcription factors, MYB118 and MYB115, also play important roles in somatic embryogenesis. These transcription factors promote the expression of *LEC1*; overexpression of both resulted in the formation of somatic embryos on root explants (Wang et al., 2008). The micro RNA miR396 is associated with somatic embryogenesis induction and helps control *PLT1* and *PLT2* (Szczygieł-Sommer and Gaj, 2019). Other evidence suggests that AGL15 forms protein complexes with SOMATIC EMBRYOGENESIS RECEPTOR-LIKE KINASES (SERKs), which are induced in response to auxin (Zheng et al., 2009). Ethylene has also been shown to impact somatic embryogenesis because interactions between ETHYLENE RESPONSE FACTOR 002 (ERF022) and LEC2, and the involvement of other AP2/ERF transcription factors have been observed (Zheng et al., 2013; Xu and Huang, 2014; Horstman et al., 2017). Reprogramming of somatic cells to form embryos and then back to differentiated somatic cells requires multiple hormone signaling pathways to work together.

Genomic DNA methylation patterns change during development. In mature tissues, *LEC1*, *LEC2*, and *AGL15* are hypermethylated in somatic cells, while hypomethylation has been seen of similar genes (e.g., *SERKs*, *LEC2*, *WUS)* in embryonic calli (Fraga et al., 2012). This suggests that somatic embryogenesis and genes involved in embryonic cell to somatic cell transition is subject to epigenetic regulation as the repression of embryonic genes leads to the development of mature and differentiated tissues. There is conflicting evidence for the role of DNA methylation in somatic embryogenesis. In some studies, the demethylation agent 5-azacitidine strongly inhibited embryogenesis in *Medicago truncatula* and *Arabidopsis* (Santos and Fevereiro, 2002; Grzybkowska et al., 2018), while in other plants, such as in *Coco nucifera* and *Acca sellowiana*, its application increased embryogenesis (Fraga et al., 2012; Osorio-Montalvo et al., 2020). This suggests that differential DNA methylation is required for successful somatic embryogenesis but its effects are highly genotype and species dependent.

Two critical regulatory epigenetic pathways, PcG and PKL, are involved in the epigenetic regulation of genes during somatic embryogenesis. As in shoot organogenesis, the PRC2-mediated H3K27 methylation, part of the PcG pathway, is involved in the repression of *LEC1*, *LEC2*, and *FUS3*, aiding in the transition from embryonic to somatic cells (Makarevich et al., 2006). The Repressive LEC2 Element (RLE) in the *LEC2* promoter recruits PCR2 for methylation and repression of *LEC2* in somatic cells (Berger et al., 2011). Evidence supporting this includes an increase in somatic embryogenesis of *Arabidopsis* in vegetative tissue depleted of PRC2 (Mozgová et al., 2017). PRC2 has also been shown to negatively regulate other important regulators of plant regeneration including WOX5, WOX11, WUS, and STM. PKL encodes for a chromatin remodeling factor, which serves to rearrange nucleosome positions in order to regulate gene expression. Multiple studies have demonstrated that *pkl* mutants show an increase in the ectopic expression of *LEC1*, *LEC2*, and *FUS3*, resulting in embryonic traits in somatic tissues (Ogas et al., 1997; Henderson et al., 2004). This suggests that PKL plays a role in negatively regulating embryonic genes in somatic tissues. However, the specific molecular mechanism by which PKL works is still unclear.

### Small Signaling Peptides in Plant Regeneration

Signaling peptides are important players in plant development. One family of signaling peptides, CLAVATA/ENDOSPERM SURROUNDING REGION (CLE), has central roles in modulating stem cell differentiation during plant growth and development (Katsir et al., 2011). These peptides are post-translationally processed and contain a signal peptide targeting the protein for secretion, where it is used for cell-to-cell communication (Yamaguchi et al., 2016). In *A. thaliana*, there are 32 CLE peptides including CLV1, CLV2, and CLV3. CLV3 is secreted from cells and interacts with CLV1, a leucine-rich repeat receptor kinase, to maintain stem cell populations in the apical meristem (Clark et al., 1995; Hirakawa et al., 2008). In *clv1* and *clv3* mutants, plants develop enlarged shoot and floral apical meristems, suggesting improper signaling disruption to maintenance of stem cell populations (Clark et al., 1995). *WUS* promotes cell proliferation and division and upregulates *CLV1*-*CLV3*. This results in the downregulation of *WUS* by CLV1-CLV3 in a negative feedback loop. This feedback mechanism produces and maintains a constant stem cell pool (Mayer et al., 1998; Brand et al., 2000). Manipulating either WUS, CLV1, and/or CLV3 could lead to larger stem cell pools and greater potential for cell division. This in conjunction with downstream molecular players, such as *CUC* genes, *PLT* genes, or *SPL*, and could potentially lead to more and faster plant regeneration. However, this would require careful orchestration of the key regulators.

### Growth Regulating Factors as Agents for Increased Regeneration

Growth Regulating Factors (GRF) are a transcription factor family that regulates many aspects of plant growth and development including leaf, stem, root, seed development, flowering, regulation of stress, and plant longevity. The first GRF, Os-*GRF1*, was identified two decades ago during a differential expression study of responses of deep-water rice to GA (van der Knaap et al., 2000). GRFs have now been identified in many plant species, where typically 8–20 different GRF genes are present in each genome (Omidbakhshfard et al., 2015). GRFs form complexes with their cofactor, GRF-interacting Factors (GIF), and will bind to *cis-*regulatory elements of different developmental genes in plants (Kim, 2019). For example, *AtGRF7* binds to the promoter of the AP2/ERF gene *Dehydration responsive element binding protein2A* (*DREB2A*) and represses gene expression in leaf veins (Kim et al., 2012). In *Arabidopsis, GRFs* have been shown to be expressed in leaf and root tissue where prolific cell growth is occurring and tend to decrease with plant age (Kim et al., 2003; Lee et al., 2009; Hewezi et al., 2012; Szczygieł-Sommer and Gaj, 2019).

GRF proteins are post-transcriptionally regulated by miR396 throughout the course of plant development; miR396 recognizes and binds to GRF, resulting in degradation or translational arrest. Expression of miR396 occurs at low levels in leaf primordia that gradually increase throughout organ development and maturity (Rodriguez et al., 2010). Expression of *AtGRF2* is restricted to specific portions of the leaf during development through antagonistic expression of *miR396* (Rodriguez et al., 2010). In rice, *miR396* mutants resulted in an upregulation of multiple *GRF* genes, in particular *GRF3*. These mutants also produced plants with longer leaves and shorter internodes (Miao et al., 2020). Because of their involvement in organ development, GRF and miR396 are potential targets for increasing *in vitro* regeneration.

GRFs regulate players important for *in vitro* regeneration. GRF proteins from rice, OsGRF3 and OSGRF10 repress promoter activity of a KNOX gene, *Oskn2* (Kuijt et al., 2014). In the same study, barley GRF, BGRF1, repressed *Hooded/Bkn3*, a barley KNOX gene, and overexpression of *OsGRF10*, *AtGRF4*, *AtGRF5*, and *AtGRF6* repressed activity of *KNAT2* in *Arabidopsis*. In addition, overexpression of *OsGRF3* and *OsGRF10* induced root and shoot formation on primary tillers of rice (Kuijt et al., 2014). Because regulation of *KNOX* genes is necessary for cell identity transitions from meristem cells to mature organ cells (Hake et al., 1995, 2004; Tsuda et al., 2011), the reported functions of these GRFs demonstrate the potential importance of GRFs in both organogenesis and somatic embryogenesis. An RNA-seq study in rice showed upregulation of *OsGRF6* resulted in an increase in expression of the auxin biosynthesis gene, *OsYUCCA-like*, and signaling genes, *OsARF2*, *OsARF7*, *OsARF11* (Gao et al., 2015). In addition, altered expression of *GRF* and *GIF* affect root growth through regulation of *PLT1*, *PLT2*, and *SCR* (Ercoli et al., 2018). In Arabidopsis, the double mutant *gif1/an3 gif2* and the triple mutant *gif1/an3 gif2 gif3* both showed the formation of a disorganized QC and larger RAM, while overexpression of *GRF3* with a mutated miRNA binding site (*rGRF3*) resulted in smaller meristems (Ercoli et al., 2018). These studies provide evidence that GRFs and GIFs are upstream regulators of molecular determinants involved in callus formation and shoot meristem identity, giving altered expression of GRFs and GIFs the potential to increase regeneration in plants.

GRFs and GIFs have now been shown to enhance regeneration capacity and rates in plants. Ectopic expression of *AtGRF5* and orthologs increased callus production in canola and shoot organogenesis in sugar beet, soybean, and sunflower; also, ectopic expression of the maize *GRF5* ortholog increased formation of embryogenic calli indicating that GRFs regulate multiple *in vitro* regeneration pathways (Kong et al., 2020). In addition, transformation with a chimeric *GRF-GIF* gene fusion can increase the rate and number of regenerates in wheat, rice, and citrus (Debernardi et al., 2020). Independent transformations and co-transformations of multiple wheat *GRF*s fused with *GIF*s were studied including *GRF4*, *GRF5*, *GIF1*, *GIF2*, and *GIF3*; the chimeric transgene composed of a fusion between GRF4 and GIF1 (GRF4-GIF1) resulted in the highest frequency of regeneration in wheat among all combinations of GRFs and GIFs tested. In addition to increased regeneration, shoot regeneration and transgenesis in wheat was successful without the use of cytokinins in the culture medium. Furthermore, regeneration could be induced from leaf explants rather than immature embryos. The efficacy of chimeric transgene was also tested in the dicotyledonous species, *Citrus*, using the *Citrus* and *Vitis* GRF4 and GIF1 homologs (Debernardi et al., 2020). Furthermore, the use of the microRNA insensitive *rGRF4-GIF* resulted in greater stimulation of regeneration in wheat, rice, and *Citrus*. This is a major breakthrough and will be exploited for the regeneration of recalcitrant species and cultivars, leading to a likelihood of higher transformation rates.

## Prospects for Enhanced Regeneration in Lettuce

### Synopsis of Studies on the Regeneration of Lettuce

**FIGURE 4:**
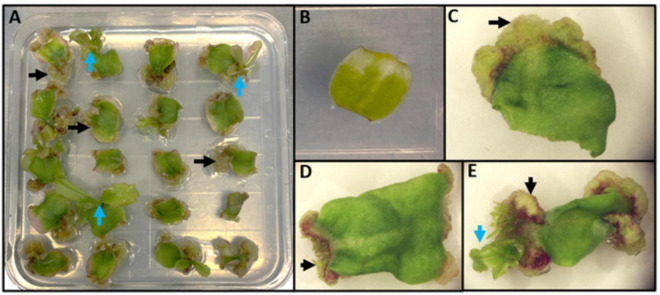
Representation of indirect *de novo* shoot organogenesis in lettuce. **(A)** A plate of 20 explants undergoing indirect *de novo* shoot regeneration. Black arrows represent friable callus formation at the wounded end of explants; blue arrows represent shoot regeneration from calli. **(B)** An explant before callus formation. **(C)** An explant during callus formation (black arrow). **(D)** First organized growth from callus (black arrow). **(E)** Indirect shoot regeneration (blue arrow) from callus (black arrow).

Lettuce, *Lactuca sativa* L. (Compositae), is a dicotyledonous plant that can be regenerated by indirect *de novo* shoot organogenesis (Figure 4) and was a model for early studies of regeneration (reviewed in Michelmore and Eash, 1985). Some genotypes regenerate readily on a variety of media formulations and growth regulators; however, some lettuce genotypes are recalcitrant to regeneration. Lettuce is also amenable to *Agrobacterium*-mediated transformation (Michelmore et al., 1987). Protocols for high efficiency, genotype-independent regeneration of lettuce are required in order to fully benefit from biotechnological approaches, including genome editing, for crop improvement. Given differences in regeneration rates of different genotypes and the wealth of knowledge from model species described above, top-down and bottom-up approaches to the molecular basis of regeneration in lettuce could lead to protocols for enhanced regeneration of multiple genotypes.

Lettuce regeneration has been studied for many decades. Lettuce was among the first plants to be tested for regeneration. The first studies on *in vitro* regeneration of lettuce failed to produce shoots from leaves of *L. sativa* and *L. canadensis* (LaRue, 1933, 1936). Later, regeneration of lettuce shoots was successful with the addition of adenine and kinetin to the growth medium (Doerschug and Miller, 1967). In this study, the regenerative capability of hypocotyl, cotyledon, and mature leaf explants was tested on the same base medium with different combinations of IAA, kinetin, and adenine, and cotyledons were shown to be the most effective explant source for shoot regeneration. In the same study, kinetin was effective at promoting the transition from callus formation to shoot regeneration (Doerschug and Miller, 1967). This suggested that in lettuce, as shown in other plant species, high levels of cytokinin promotes the formation of shoot meristems that results from the transition of cell fate from root-like callus cells to shoot cells. Later studies focused on the optimization of factors influencing lettuce regeneration, including media formulations, plant growth regulator use, light requirements, temperature, explant type, and genotype (Doerschug and Miller, 1967; Kadkade and Seibert, 1977; Koevary, 1978; Sasaki, 1979, 1982; Alconero, 1983; Webb et al., 1984; Michelmore and Eash, 1985). Light intensity and photoperiod were shown to be also important for lettuce regeneration; cotyledon explants developed well-formed shoots with a 16-h photoperiod but significantly fewer shoots formed in the dark; additionally, the presence of red light doubled the number of buds and shoots (Kadkade and Seibert, 1977). In aggregate, callus formation occurred on all lettuce cultivars studied when using both auxins and cytokinins in the culture medium, although there were differences between genotypes. Shoot regeneration was elicited when the medium contained cytokinins with little or no auxins. Although mature leaves and hypocotyls showed regenerative capabilities, cotyledons were the most amenable explant source for regeneration.

Indirect *de novo* shoot organogenesis in lettuce involves cell divisions of spongy, palisade, and epidermal cells. A cytohistological study of adventitious bud formation from cotyledon explants revealed initial divisions of spongy and palisade cells followed by divisions of epidermal cells to form tetrads (Nuti Ronchi and Gregorini, 1970). Callus was formed from the division of mesophyll cells and inward proliferation of epidermal cells. Subsequently, adventitious buds arose from one or two epidermal cells, which led to the formation and organization of shoot apical meristems. This study provided the timeline and steps that occur during organogenesis; however, the tools were not available to study the underlying genetic and molecular constituents responsible for the changes in cell anatomy and transition of cell fate, particularly epidermal cells to meristematic centers.

Like most plant species, regenerative capacity is highly dependent on genotype and there is considerable variation in regenerative capacity among lettuce cultivars (Michelmore et al., 1987; Curtis et al., 1994; Ampomah-Dwamena et al., 1997; Mohebodini et al., 2011). There is no significant correlation to regeneration efficiency and morphological group (i.e., crisphead, butterhead, cos, and leaf). In a side-by-side study, highly regenerating genotypes included Bambino (crisphead), Iceberg (crisphead), Cobham Green (butterhead), Sweet Butter (butterhead), Simpson Elite (leaf), Rosalita (cos), and Paris White (cos); recalcitrant genotypes included Oak Leaf (leaf), Royal Oak Leaf (leaf), Sangria (crisphead), and Mainspring (butterhead) (Ampomah-Dwamena et al., 1997). Generation of stable transgenics of lettuce relies on *Agrobacterium*-mediated transformation and *in vitro* regeneration. Therefore, it is important to understand and identify the genetic and molecular players to increase regeneration in order to manipulate recalcitrant lettuce varieties.

### Known Molecular Determinants for Regeneration in Lettuce

There have been few studies on the molecular determinants of regeneration in lettuce. A dominant mutation of the ethylene receptor ETR1-1 was shown to inhibit shoot regeneration in lettuce (Kim and Botella, 2004). Lettuce cultivars LEI26 and Seagreen were transformed using *Agrobacterium-*mediated transformation for the introduction of GUS under the control of the CaMV 35S constitutive promoter and the mutated ethylene receptor *etr1-1* under the control of a leaf senescence-specific promoter, *sag12.* Transformations with 35S:GUS showed high regenerative potential with 85% of explants developing shoots, while the introduction of *sag12:etr1-1* significantly reduced regenerative potential with only 2.86% of explants producing shoots. Explants transformed with *sag:etr1-1* also stimulated root formation directly from cotyledon explants without the formation of callus (Kim and Botella, 2004). This suggests that ethylene responses are important in *in vitro* lettuce regeneration in which inhibiting ethylene receptors promotes root formation and inhibits callus and shoot formation. This is consistent with observations of other ethylene response factors during *in vitro* regeneration, such as the early expression of AP2/ERF transcription factors during callus formation and the involvement of ERF022 activity during somatic embryogenesis (Iwase et al., 2011a,b; Zheng et al., 2013; Xu and Huang, 2014; Horstman et al., 2017).

Data is limited for lettuce on the effects of the pathways and molecular determinants described in other species. A recent study examined the chronological expression of homeobox genes during *in vitro* regeneration of lettuce (Farina et al., 2021). Gene expression profiles of lettuce homologs to the homeobox WOX family transcription factor genes *WUS* (*LsWUS1L* and *LsWUS2L*) and the KNOTTED1-LIKE homeobox family transcription factor gene *ST-M* (*LsSTM*), were examined in cotyledon explants over 12 days on inductive medium. A time course analysis showed a steady increase of expression of *LsWUS1*; in early days of culture, increased expression of *LsWUS2L* correlated with the formation of poorly vacuolated cells with large nuclei in the explants. Expression of *LsSTM1L* also drastically increased in early days of culture, followed by a later decrease, suggesting that it helps recruit proteins and regulates expression of genes needed for the initiation of regeneration in lettuce (Farina et al., 2021). This parallels patterns of *WUS* and *STM* expression observed early in plant regeneration, specifically during the formation of shoot promeristems and meristematic centers from callus in *Arabidopsis* (Daimon et al., 2003; Zhang et al., 2017). This is also consistent with the essential role WUS plays in maintaining the stem cell pool that is critical for proper SAM function (Sarkar et al., 2007). The CCAAT-binding transcription factors, *LEC1* and *LEC2*, play a major role in development and maturation of embryos (see sections “Embryogenic Callus Formation and Somatic Embryogenesis”). Nothing has been reported for homologs of *LEC1* and *LEC2* in lettuce. It would be interesting to overexpress homologs of these transcription factors in lettuce to determine if this results in enhanced regeneration as in *Arabidopsis*, tobacco, and cassava (Gaj et al., 2005; Guo et al., 2013; Brand et al., 2019). Similarly, over-expression of *CUC1* and *CUC2* as well as *PLT* genes (see sections “Organogenic Callus Formation, *De novo* Root Organogenesis, and *De novo* Shoot Organogenesis”) may also result in enhanced regeneration of lettuce as in Arabidopsis (Ikeda et al., 2006; Matsuo et al., 2009; Kareem et al., 2015).

### MADS-Box Genes in Lettuce

MADS-box transcription factors, particularly AGL15 and AGL18, are major molecular players involved in *in vitro* regeneration (see sections “Embryogenic Callus Formation and Somatic Embryogenesis”). There are at least 82 MADS-box encoding genes in lettuce (Ning et al., 2019), most of which have been studied in relation to flowering time and floral development (reviewed in Han et al., 2021). Of these 82 genes, 23 encoded for M-type genes of the type 1 lineage and 59 floral genes of the type II lineage containing a MIKC domain. Within the type II MADs-box genes, 10 belonged to the *AGL15* subfamily which contained homologs of Arabidopsis and tomato *AGL15* genes. Currently, no work has been reported on the role of lettuce MADs-box genes during *in vitro* regeneration. The 10 genes identified in the *AGL15* subfamily should be characterized for their roles in regeneration in lettuce; it should be tested whether over expression of ALG15 results in increased somatic embryogenesis as in *Arabidopsis* (Paul et al., 2022).

### Growth Regulating Factors in Lettuce

There are 15 *GRF* genes in lettuce and their chromosomal locations, gene structure, conserved motifs, and expression patterns have been characterized (Zhang et al., 2021). One *GRF* gene was studied in detail. *LsaGRF5* showed low expression in leaves and roots with high expression in reproductive buds, suggesting an important function in flower development. The GRF regulator, *miR396a*, had high expression in mature flowers and stems and low expression in reproductive buds. These data suggest that high levels of *LsaGRF5* expression in young tissues is coincident with actively dividing cells; as the cells and tissues mature, *LsaGRF5* becomes downregulated by *miR396a*; this is similar to what is observed in other species (see section “Growth Regulating Factors as Agents for Increased Regeneration”). Furthermore, overexpression of *LsaGRF5* resulted in larger leaf size, while overexpression of miR396a resulted in smaller leaf size (Zhang et al., 2021). However, none of the 15 *GRF* genes have been characterized for their effects on regeneration in lettuce. Given the success of GRF or GRF-GIF fusions with other species (see section “Growth Regulating Factors as Agents for Increased Regeneration”), it is likely that similar enhanced rates of regeneration and transformation will be reported soon.

## Conclusion and Future Perspectives

The underlying processes of plant regeneration all involve cell fate transition by reprogramming gene expression. The several pathways involved in plant development and regeneration are complex. Although each pathway has unique molecular players, many of the key regulators overlap and have important functions in each. Auxin and cytokinin signaling pathways play a major role in regulating multiple regenerative pathways and accompany the genome-wide switch in gene expression profile during the early stages of regeneration. Other phytohormones, such as GA, ABA, and ethylene, also contribute to plant regeneration and cell fate transition.

Many of the players and regulators important for *in vitro* regeneration have been studied in model species, such as *Arabidopsis*, but have not been functionally characterized in non-model species such as lettuce. The complete genome sequence of *L. sativa* (Reyes-Chin-Wo et al., 2017) has provided useful genic targets for modification by genome editing. Currently, genome editing of lettuce requires *Agrobacterium*-mediated transformation, which requires *in vitro* regeneration; therefore, studying molecular determinants and understanding pathways controlling regeneration in lettuce has great value. Identifying orthologs of genes discussed in this review and then characterizing them in other systems, such as lettuce, will help form a more generalized understanding of *in vitro* regeneration in plants. Further studies on identification of recalcitrant varieties, quantitative trait locus analyses on varieties with varying regenerative capabilities, and expression profiles during *in vitro* regeneration could provide insight into other genes regulated during *in vitro* regeneration of lettuce. Understanding these pathways in lettuce will allow for a better understanding of the pathways in other important crops, particularly within the Compositae family such as sunflower, artichoke, safflower, and many ornamentals.

## Author Contributions

RM and TB conceived the idea for the manuscript. TB conducted the literature review and drafted the manuscript and figures. Both authors reviewed the final manuscript and approved submission.

## Conflict of Interest

The authors declare that the research was conducted in the absence of any commercial or financial relationships that could be construed as a potential conflict of interest.

## Publisher’s Note

All claims expressed in this article are solely those of the authors and do not necessarily represent those of their affiliated organizations, or those of the publisher, the editors and the reviewers. Any product that may be evaluated in this article, or claim that may be made by its manufacturer, is not guaranteed or endorsed by the publisher.
